# Genome sequence and description of the mosquitocidal and heavy metal tolerant strain *Lysinibacillus sphaericus* CBAM5

**DOI:** 10.1186/1944-3277-10-2

**Published:** 2015-01-20

**Authors:** Tito David Peña-Montenegro, Lucía Lozano, Jenny Dussán

**Affiliations:** 1 Centro de Investigaciones Microbiológicas – CIMIC, Universidad de los Andes, Bogotá, Colombia

**Keywords:** *Lysinibacillus sphaericus* CBAM5, DNA homology, Binary toxins, Mosquitocidal toxins, S-layer proteins, Heavy metal tolerance

## Abstract

*Lysinibacillus sphaericus* CBAM5, was isolated from subsurface soil of oil well explorations in the Easter Planes of Colombia. This strain has potential in bioremediation of heavy-metal polluted environments and biological control of *Culex quinquefasciatus.* According to the phylogenetic analysis of 16S rRNA gene sequences, the strain CBAM5 was assigned to the *Lysinibacillus sphaericus* taxonomic group 1 that comprises mosquito pathogenic strains. After a combination assembly-integration, alignment and gap-filling steps, we propose a 4,610,292 bp chromosomal scaffold. The whole genome (consisting of 5,146,656 bp long, 60 contigs and 5,209 predicted-coding sequences) revealed strong functional and syntenial similarities to the *L. sphaericus* C3-41 genome. Mosquitocidal (Mtx), binary (Bin) toxins, cereolysin O, and heavy metal resistance clusters from *nik, ars, czc, mnt, ter, cop, cad,* and *znu* operons were identified.

## Introduction

*Lysinibacillus sphaericus* is one of the bacteria used as a bio-insecticide as part of vector control programs against tropical diseases, such as malaria, filariasis, yellow fever, dengue fever and West Nile virus [1]. *L. sphaericus* isolates may be classified according to their larvicidal activity into high and low toxicity strains. High- and low-toxicity strains synthesize mosquitocidal toxins (Mtx) in vegetative growth cells [2]. Highly toxic strains produce a binary toxin coded by *binA* and *binB* genes in sporulating stages [3]. In addition, *L. sphaericus* larvicidal toxicity may be explained due to expression of Cry48/Cry49 toxin [4] and the S-layer protein [5]. Vegetative and sporulated cells of *L. sphaericus* CBAM5 are pathogenic towards *Culex quinquefasciatus* larvae [6]. LC50 (50% lethal concentration) toward *C. quinquefasciatus* larvae of strain CBAM5 is 1400 cells/mL from sporulated cultures, being this isolate assigned as a high-toxicity strain [6].

The biotechnological application of *L. sphaericus* is not limited to biological control. *L. sphaericus* biomass has been applied in the bioremediation of heavy metals, such as cobalt, copper, chromium and lead [7] with specific metal binding in the cell surface [8]. Native Colombian isolates *L. sphaericus* OT4b.31 and IV(4)10 showed heavy metal biosorption in living and dead biomass, both strains expressing the S-layer proteins [9]. *L. sphaericus* strain CBAM5, along with other 24 native isolates, shown effective growth in arsenate, hexavalent chromium and/or lead [6,10].

Considering that *Lysinibacillus sphaericus* CBAM5 is a relevant native strain, not only by its highly toxic larvicidal activity but also by its heavy metal tolerance, we have chosen this strain to analyze its genomic sequence. In this report, we present a summary classification, and set of general features for *Lysinibacillus sphaericus* strain CBAM5 including previously unreported aspects of its phenotype, together with the description of its genome sequence and annotation.

### Organism information

*Lysinibacillus sphaericus* is an aerobic, mesophilic, spore-forming and Gram-positive bacterium, commonly isolated from soil and water [11]. Formerly known as *Bacillus sphaericus,* the species was later reassigned to the genus *Lysinibacillus* because of its distinctive peptidoglycan membrane composition, and physiological features [12,13]. *Lysinibacillus sphaericus* strains have been classified into five DNA homology groups, where mosquito larvicidal strains were placed into DNA subgroup IIA [14] while the subgroup IIB was reclassified as *Lysinibacillus fusiformis*[15]. Later, according to 16S rDNA and lipid profile comparisons, *Lysinibacillus sphaericus* strains were classified into seven similarity subgroups, of which only four retained the previous description by Krych et al. [15]. Groups VI and VII were later reclassified as new species [16]. Because of the phenotypic and genetic diversity summarized above, most of the groups remain designated as *Lysinibacillus sphaericus sensu lato.*

**Figure 1:**
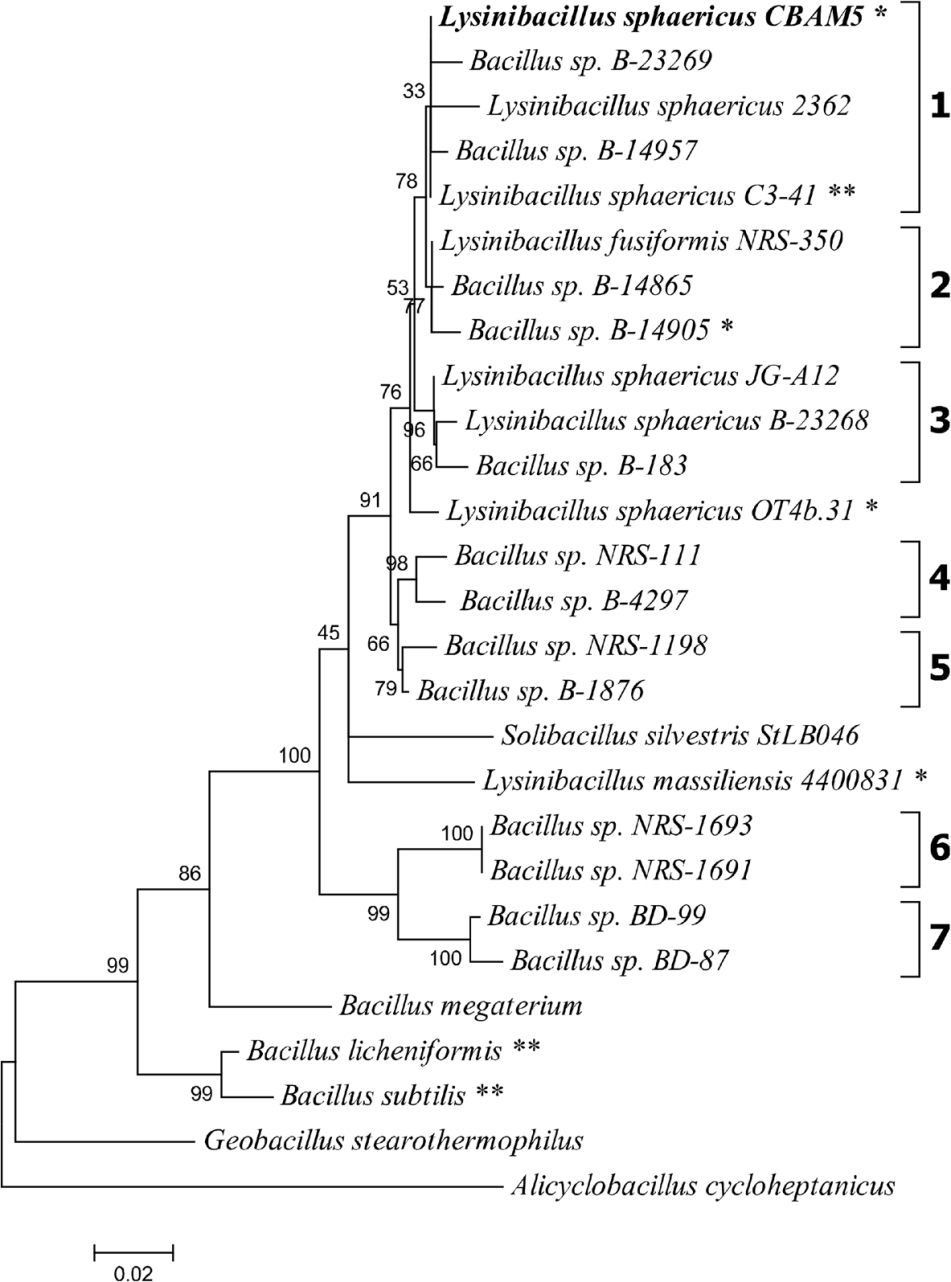
**Phylogenetic tree highlighting the position of *Lysinibacillus sphaericus *CBAM5.** Phylogenetic analyses included available type strains and other non-assigned species within the families *Alicyclobacillaceae* and *Bacillaceae*. Right brackets encompass each homology group (1–7) according to Nakamura’s benchmarks [15]. Nucleotide sequences obtained from GenBank and used in the phylogenetic analyses were as follows: *Alicyclobacillus cycloheptanicus* 1457 (X51928), *Geobacillus stearothermophilus* 10 (X57309), *Bacillus subtilis* 168^T^ (X60646), *Bacillus licheniformis* DSM 13^T^ (X68416), *Bacillus megaterium* IAM 13418^T^ (D16273), *Bacillus sp.* BD-87 (AF169520), *Bacillus sp.* BD-99 (AF169525), *Bacillus sp.* NRS-1691 (AF169531), *Bacillus sp.* NRS-1693 (AF169533), *Solibacillus silvestris* StLB046 (NR_074954), *Lysinibacillus massiliensis* 4400831 (NR_043092), *Bacillus sp.* B-1876 (AF169494), *Bacillus sp.* NRS-1198 (AF169528), *Bacillus sp.* B-4297 (AF169507), *Bacillus sp.* NRS-111 (AF169526), *Lysinibacillus sphaericus* OT4b.31 (JQ744623), *Lysinibacillus sphaericus* B-23268^T^ (AF169495), *Bacillus sp.* B-183 (AF169493), *Lysinibacillus sphaericus* JG-A12 (AM292655), *Bacillus sp.* B-14905 (AF169491), *Bacillus sp.* B-14865 (AF169490), *Lysinibacillus fusiformis* NRS-350 (AJ310083), *Lysinibacillus sphaericus* C3-41 (CP000817:16818–18361), *Bacillus sp.* B-14957 (AF169492), *Lysinibacillus sphaericus* 2362 (L14011), *Bacillus sp.* B-23269 (AF169496), *Lysinibacillus sphaericus* CBAM5 (KK037167:893906–895445). The tree with the highest log likelihood (-6732.2703) is shown. The percentage of trees in which the associated taxa clustered together is shown next to the branches. The tree is drawn to scale, with branch lengths measured in the number of substitutions per site. Lineages with type strain genome sequencing projects registered in GOLD [57] are labeled with one asterisk, those also listed as ‘Complete and Published’ with two asterisks.

Partial 16S rRNA gene sequences (1,421 bp) were aligned to establish the phylogenetic neighborhood of *Lysinibacillus sphaericus* CBAM5 (Figure 1). The phylogenetic tree was constructed by using the Maximum Likelihood method on the Tamura-Nei model [17]. Initial tree for the heuristic search was obtained by applying the Neighbor-Joining method to a matrix of pairwise distances estimated using the Maximum Composite Likelihood (MCL) approach. Evolutionary analyses were conducted in MEGA6 [18]. The stability of relationships was assessed by bootstrap analysis based on 1,000 resamplings for the tree topology. *L. sphaericus* CBAM5 was assigned to the DNA similarity group 1 (formerly known as DNA homology group IIA), in line with a previous classification of mosquito pathogenic native strains [6].

*Lysinibacillus sphaericus* CBAM5 was isolated from drilling mineral base oil samples (CBAM by its acronym in Spanish), collected in the Eastern Planes region in Colombia. The strain was described as an aerobic, motile, catalase positive, Gram variable rod [10]. *L. sphaericus* CBAM5 is able to grow in acetate as sole carbon source, but not in glucose (Table 1, Additional file 1: Table S1). Spherical terminal spores within swollen sporangia were observed under light microscopy (Additional file 2: Figure S1). By scanning electron microscopy, *L. sphaericus* CBAM5 is estimated to measure 0.52 to 0.60 μm in width and 2.12 to 3.11 μm long (Additional file 3: Figure S2). Cultures grow at 15 to 40°C over a pH range of 6.0 to 9.0. Antibiotic resistance was evaluated separately by adding filter sterilized antibiotic solutions in Luria-Bertani broths and checking turbidity after 15 hours of growth. *L. sphaericus* CBAM5 is sensitive to kanamycin (12.5 μg/mL), chloramphenicol (30 μg/mL), erythromycin (25 μg/mL), and gentamicin (15 μg/mL), while it showed resistance to trimethoprim/sulfamethoxazol up to 50 μg/mL/250 μg/mL.

**Table 1:** **Classification and general features of *Lysinibacillus sphaericus *** **CBAM5 according to the MIGS recommendations**[19]

**MIGS ID**	**Property**	**Term**	**Evidence code**^**a**^
	Current classification	Domain *Bacteria*	TAS [20]
		Phylum *Firmicutes*	TAS [21-23]
		Class *Bacilli*	TAS [24,25]
		Order *Bacillales*	TAS [26,27]
		Family *Bacillaceae*	TAS [26,28]
		Genus *Lysinibacillus*	TAS [13,29]
		Species *Lysinibacillus sphaericus*	TAS [11,13]
		Strain CBAM5	TAS [10]
	Gram stain	Positive in vegetative cells, variable in sporulating stages	IDA
	Cell shape	Straight rods	IDA
	Motility	Motile	IDA
	Sporulation	Sporulating	IDA
	Temperature range	15 – 40°C	IDA
	Optimum temperature	30°C	IDA
	Carbon source	Complex carbohydrates	TAS [10]
	Energy metabolism	Heterotroph	TAS [10]
MIGS-6	Habitat	Subsurface soil	TAS [10]
MIGS-6.3	Salinity	Growth in Luria-Bertani broth (5% NaCl)	IDA
MIGS-22	Oxygen requirement	Aerobic	TAS [10]
MIGS-15	Biotic relationship	Free living	TAS [10]
MIGS-14	Pathogenicity	Pathogenic toward *Culex quinquefasciatus* larvae	TAS [6]
MIGS-4	Geographic location	Eastern Planes oil basins, Colombia	TAS [10]
MIGS-5	Sample collection time	January 2005	TAS [10]
MIGS-4.1	Latitude	5.0121944	TAS [10]
MIGS-4.2	Longitude	-72.7109167	TAS [10]
MIGS-4.3	Depth	20 m	TAS [10]
MIGS-4.4	Altitude	350 m above sea level	TAS [10]

^a^Evidence codes - IDA: Inferred from Direct Assay; TAS: Traceable Author Statement (i.e., a direct report exists in the literature); NAS: Non-traceable Author Statement (i.e., not directly observed for the living, isolated sample, but based on a generally accepted property for the species, or anecdotal evidence). These evidence codes are from the Gene Ontology project [30].

### Genome sequencing information

#### Genome project history

The genome sequencing of *Lysinibacillus sphaericus* CBAM5 was supported by the CIMIC (Centro de Investigaciones Microbiológicas) laboratory at the University of Los Andes within the Grant (1204-452-21129) of the Instituto Colombiano para el Fomento de la Investigación Francisco José de Caldas. Whole genomic DNA extraction and bioinformatics analysis was performed at CIMIC laboratory, whereas libraries construction and whole shotgun sequencing at the Beijing Genome Institute (BGI) Americas Laboratory (Tai Po, Hong Kong). The applied pipeline included quality check of reads, de novo assembly, a gap-filling step and mapping against a reference genome. This whole genome shotgun project has been deposited at DDBJ/EMBL/GenBank under the accession AYKQ00000000. The version described in this paper is the first version, AYKQ01000000. A summary of the project information is shown in Table 2.

**Table 2:** Genome sequencing project information

**MIGS ID**	**Property**	**Term**
MIGS-31	Finishing quality	Improved high-quality draft
MIGS-28	Libraries used	One paired end tags 90:90 bp with 500 bp insert
MIGS-29	Sequencing platforms	Illumina Hi-Seq 2000
MIGS-31.2	Fold coverage	100×
MIGS-30	Assemblers	CISA version 1.3, SOAPdenovo version 2.04, Velvet version 1.2.10, ABySS version 1.3.7, CLC Assembly Cell version 4.0.10
MIGS-32	Gene calling method	Glimmer3, tRNAscan-SE, RNAmmer
	Genbank ID	AYKQ00000000
	Genbank Date of Release	February 1, 2014
	GOLD ID	Gi0057485
	Project relevance	Biotechnology, metabolic pathway

### Growth conditions and DNA isolation

*Lysinibacillus sphaericus* strain CBAM5 was grown in nutrient broth for 16 hours at 30°C and 150 rev/min. High molecular weight DNA was isolated using the EasyDNA® Kit (Carlsbad, CA, USA. Invitrogen) as indicated by the manufacturer. DNA purity and concentration were determined in a NanoDrop spectrophotometer (Wilmington, DE, USA. Thermo Scientific).

### Genome sequencing and assembly

After DNA extraction, samples were sent to the Beijing Genome Institute (BGI) Americas Laboratory (Tai Po, Hong Kong). Purified DNA sample was first sheared into smaller fragments with a desired size by a Covaris E210 ultrasonicator. Then the overhangs resulting from fragmentation were converted into blunt ends by using T4 DNA polymerase, Klenow Fragment and T4 polynucleotide kinase. After adding an “A” base to the 3’ end of the blunt phosphorylated DNA fragments, adapters were ligated to the ends of the DNA fragments. The desired fragments were purified though gel-electrophoresis, then selectively enriched and amplified by PCR. The index tag was introduced into the adapter at the PCR stage as appropriate, and a library quality test was performed. Lastly, qualified, short, paired-ends of 90:90 bp length with 500 bp insert libraries were used to cluster preparation and to conduct whole-shotgun sequencing in Illumina Hi-Seq 2000 sequencers.

Using the FASTX-Toolkit version 0.6.1 [31] and clean_reads version 0.2.3 from the ngs_backbone pipeline [32] reads were trimmed and quality filtered. Four preliminary assemblies were obtained by using: SOAPdenovo version 2.04 [33], Velvet version 1.2.10 [34], ABySS version 1.3.7 [35], and CLC Assembly Cell version 4.0.10 [36]. Those assemblies were integrated in the CISA pipeline resulting in a consensus assembly [37]. SOAPdenovo and CLC Assembly Cell packages included automatic scaffolding and k-mer/overlapping optimization steps. To obtain structural insight of a chromosomal scaffold, we used CONTIGuator.2 [38], using the *Lysinibacillus sphaericus* strain C3-41 chromosome (accession number: CP000817.1) as reference. Some gaps were successfully filled by using GapFiller [39]. Gap-filling steps were applied over each one of the preliminary assemblies and over the final consensus assembly. Quality assessment of the assembly was performed with iCORN [40]. The error rate of the final assembly is less than 1 in 1,000,000 bp. We compared the chromosomal assembly of *L. sphaericus* CBAM5 with the chromosome sequences of *L. sphaericus* C3-41 and *L. sphaericus* OT4b.31 by maximal unique matching of translated sequences with PROmer [41], and a read mapping single nucleotide polymorphism (SNP) effect analysis with SnpEff package [42].

### Genome annotation

The Glimmer 3 gene finder was used to identify and extract sequences for potential coding regions. To achieve the functional annotation steps, the RAST server [43] and Blast2GO pipelines [44] were used. Blast2GO performed the blasting, GO-mapping and annotation steps; which included a description according to the ProDom, FingerPRINTScan, PIR-PSD, Pfam, TIRGfam, PROSITE, ProDom, SMART, SuperFamily, Pattern, Gene3D, PANTHER, SignalIP and TM-HMM databases. The results were summarized with InterPro [45]. Additionally, a GO-EnzymeCode mapping step was used to retrieve KEGG pathway-maps. tRNA genes were identified by using tRNAscan-SE [46] and rRNA genes by using RNAmmer [47]. The possible orthologs of the genome were identified based on the COG database and classified accordingly [48]. Prophage region prediction was also conducted by using the PHAST tool [49].

## Genome properties

**Figure 2:**
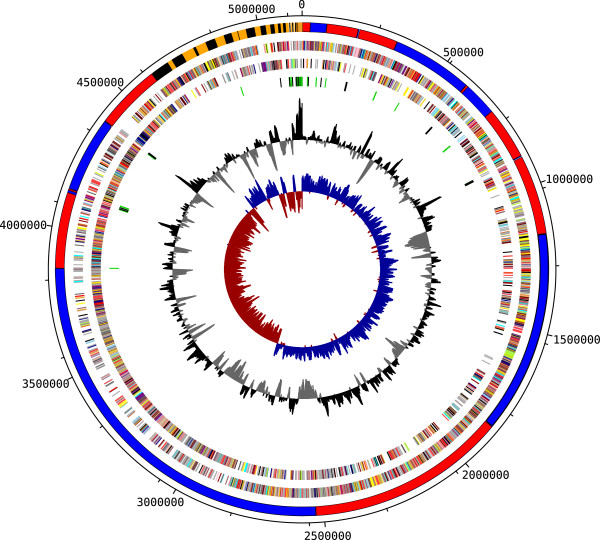
**Graphical map of the genome.** From outside to the center: Ordered and oriented scaffolds assigned to chromosome in blue and red, extrachromosomal scaffolds in orange and black, Genes on forward strand (color by COG categories), Genes on reverse strand (color by COG categories), RNA genes (tRNAs green, rRNAs gray), GC content and GC skew.

The genome summary and statistics are provided in Tables 3 and 4, and Figure 2. The genome consists of 60 scaffolds in 5,146,656 bp total size with a GC content of 37.19%. A total of 19 scaffolds were successfully aligned to a reference sequence, comprising 4,610,292 bp of sequence and are represented by the red and blue bars within the outer ring of Figure 2. Of the 5,620 genes predicted, 5,209 were protein-coding genes and 207 RNAs were identified. Genes assigned a putative function comprised 57.37% of the protein-coding genes while the remaining ones were annotated as hypothetical proteins. The distribution of genes into COGs functional categories is presented in Table 5.

**Table 3:** Summary of genome

**Label**	**Size (bp)**	**Topology**	**INSDC identifier**
Chromosomal scaffold	4,610,292	Circular	KK037167.1
Extrachromosomal elements	536,364	Linear	KK037168.1-KK037224.1

**Table 4:** Nucleotide content and gene count levels of the genome

**Attribute**	**Value**	**% of total**^**a**^
Genome size (bp)	5,146,656	100.00
DNA GC content (bp)	1,913,947	37.19
DNA coding region (bp)	4,311,603	83.77
Number of replicons	1	
Total genes	5,620	100
RNA genes	207	3.68
tRNA genes	180	3.20
Protein-coding genes	5,209	92.69
Genes in paralog clusters	151	2.69
Genes assigned to COGs	3701	65.85
1 or more conserved domains	2,520	44.84
2 or more conserved domains	834	14.84
3 or more conserved domains	361	6.42
Genes with function prediction	3,224	57.37
Genes assigned Pfam domains	3,995	71.09
Genes with signal peptides	459	8.17
Genes with transmembrane helices	1,140	20.28
CRISPR repeats	1	

^a^The total is based on either the size of the genome in base pairs or the total number of protein coding genes in the annotated genome.

**Table 5:** Number of genes associated with the 25 general COG functional categories

**Code**	**Value**	**% age**^**a**^	**Description**
J	190	3.65	Translation
A	1	0.02	RNA processing and modification
K	337	6.47	Transcription
L	193	3.71	Replication, recombination and repair
B	2	0.04	Chromatin structure and dynamics
D	39	0.75	Cell cycle control, mitosis and meiosis
V	66	1.27	Defense mechanisms
T	191	3.67	Signal transduction mechanisms
M	153	2.94	Cell wall/membrane biogénesis
N	79	1.52	Cell motility
U	23	0.44	Intracellular trafficking and secretion
O	116	2.23	Posttranslational modification, protein turnover, chaperones
C	162	3.11	Energy production and conversión
G	154	2.96	Carbohydrate transport and metabolism
E	396	7.60	Amino acid transport and metabolism
F	111	2.13	Nucleotide transport and metabolism
H	167	3.21	Coenzyme transport and metabolism
I	141	2.71	Lipid transport and metabolism
P	222	4.26	Inorganic ion transport and metabolism
Q	37	0.71	Secondary metabolites biosynthesis, transport and catabolism
R	480	9.21	General function prediction only
S	441	8.47	Function unknown
-	1508	28.95	Not in COGs

^a^The total is based on the total number of protein coding genes in the annotated genome.

## Insights into the genome

**Figure 3:**
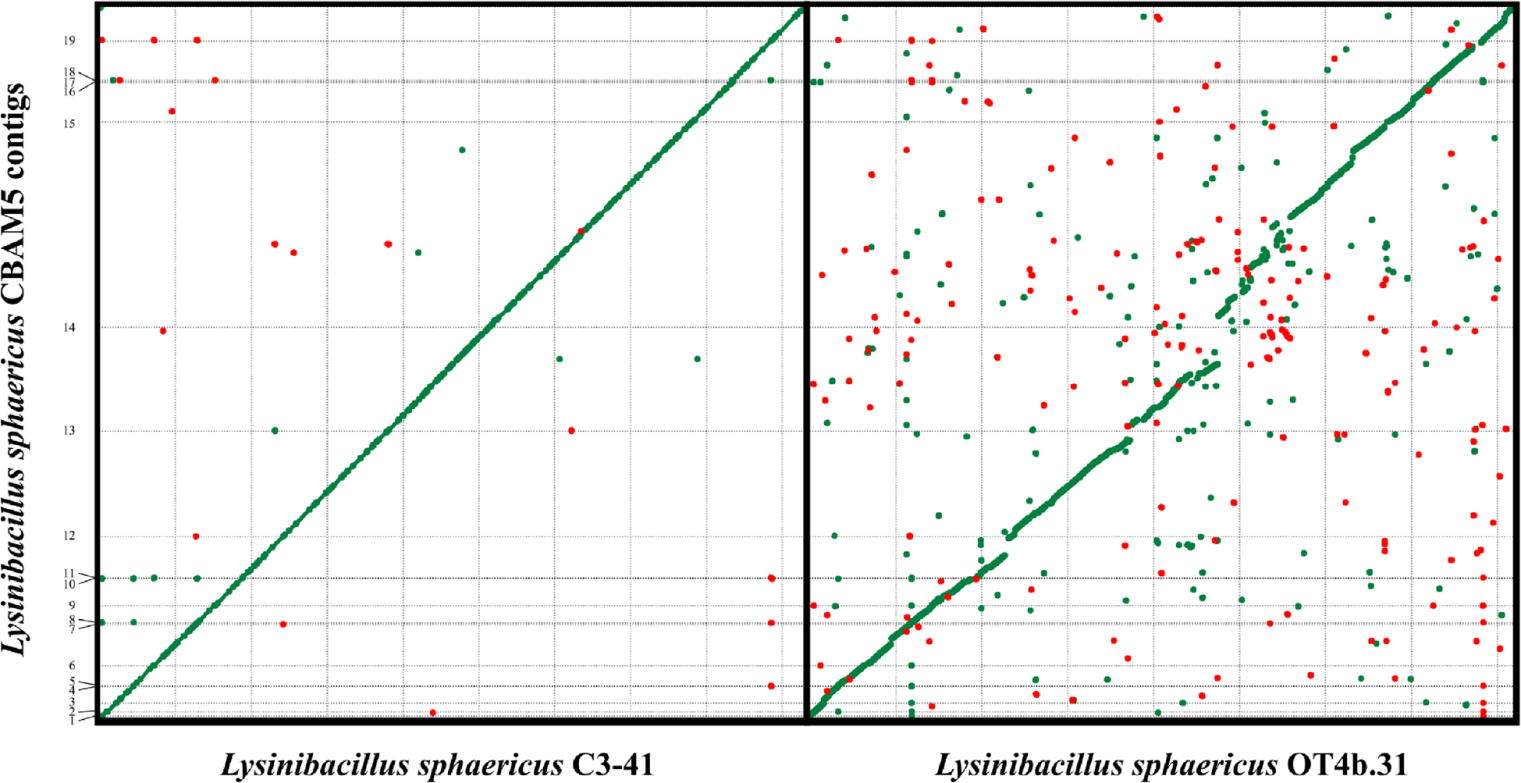
**Dot-plots of amino-acid-based alignments of *L. sphaericus *CBAM5, C3-41 and OT4b.31.** Dot-plot of amino-acid-based alignment of a 4.61 Mbp chromosomal scaffold of *L. sphaericus* CBAM5 (y-axis) to the chromosome of *L. sphaericus* C3-41 (left) and *L. sphaericus* OT4b.31 (right). Aligned segments are represented as dots or lines. Forward matches are plotted in green, reverse matches in red. Figure generated by PROmer [41].

We propose a 19-supercontig chromosomal scaffold of *Lysinibacillus sphaericus* CBAM5 with 4.61 Mbp in length, corresponding to a 99.4% of the reference chromosomal sequence. The remaining non-mapped or non-integrated contigs were aligned to plasmid reference sequences, leading to no significant coverage levels (data not shown). Then, we assigned those contigs as a set of potential extrachromosomal elements. Chromosomal comparison from the PROmer analysis between *L. sphaericus* strains CBAM5 and C3-41 showed that most of the two chromosomes mapped onto each other, revealing large segments of high similarity (Figure 3). In contrast, the comparison between the native strains *L. sphaericus* CBAM5 and OT4b.31 revealed scattered regions across the dot-plot, corresponding to low coverage levels and different synthenial arrangements. Only variants with a phred-scaled quality and depth coverage scores greater than 100 were considered valid for the SNV analysis. We found 378 variants corresponding to 4531 effects being classified as follows: 170 insertions, 280 deletions, 2020 downstream effects, 182 frame shifts, 211 intergenic effects, 2 start losts, 2 stop losts and 2114 upstream effects. In addition, no transitions, transversions, missense or silent effects were identified. As per most of the variant effects, in comparison to the C3-41 strain, are allocated upstream and downstream of the gene operons, we suggest that *L. sphaericus* CBAM5 may enclose different regulatory elements or non-coding sequences.

### Chromosome structure

The origin of replication of the chromosome of *L. sphaericus* CBAM5 was estimated by similarities to several features of the corresponding regions in *L. sphaericus* C3-41, *Bacillus* sp. B-14905 and other close related bacteria, including colocalization of the genes: *dnaA, dnaN, dnaX*, *recR* and *recF*; and GC nucleotide skew [(G–C)/(G + C)] analysis. In the contig 19 (EWH31640:EWH31645) we found a typical cluster consisting of *dnaA, dnaN, recF, gyrA* and *gyrB* boxes. The predicted genes *dnaB, dnaI, dnaG, dnaE, holA, holB, priAB, polA* and *recA* were also found spread in the chromosomal and extrachosmomal sequences. The replication termination site of the chromosomal scaffold is believed to be localized near 2.92 Mbp in the contig 14. According to GC skew analysis, the coding bias for the two strands of the chromosome is for the majority of CDSs to be on the outer strand from 0 to ~2.92 Mbp, and on the inner strand from ~2.92 Mbp to the end of the chromosomal scaffold (contig 19, Figure 2). This was also confirmed by the presence of *parC* (EWH32537) and *parE* (EWH32538), which encode the subunits of the chromosome-partitioning enzyme topoisomerase IV. Similar to previous reports [50,51], we did not find the homolog of *rtp* (replication terminator protein-encoding gene) in the chromosomal assembly of CBAM5.

### Mobile elements

*Lysinibacillus sphaericus* CBAM5 displays 28 CDSs annotated as transposases, including three allocated in the extrachromosomal sequences. The most frequent families are IS1182, IS3 and IS4. In addition, four incomplete prophage regions were identified as follows: *Thermus* phage φOH2 (contig 12), *Burkholderia* phage ST79 (contig 14), and two regions comprising the *Clostridium* phage φSM101 (contigs 14 and 28). Prophage regions φOH2 and ST79 included putative encoding sequences for tail, lysis and baseplate proteins. None of the reported phages has been described in the Colombian strain *L. sphaericus* OT4b.31 [50].

### Larvicidal toxins

The genome of *L. sphaericus* CBAM5 shows a wide repertoire of potential encoding sequences in terms of mosquitocidal toxins. In the contig 11, we found Mtx1 (EWH35097) and Mtx2 (EWH35034) CDSs located in an identical cluster as Hu et al. [51] described in the genome of *L. sphaericus* C3-41. This cluster includes two insertion sequences, one of them consisting of a disrupted transposase between the *mtx1* and *mtx2*, as well*.* One Mtx3 CDSs (EWH32377) was found in contig 14. Upstream of this sequence, we could identify some IS3 family mobile elements and putative DeoR family transcriptional regulators. In addition, we found one hypothetical toxin from the family Mtx2 (PFam PF03318) in contig 11 (EWH35106) and a putative cereolysin O CDS (EWH31995) being described to be active against the German cockroach *Blattela germanica*[52] in contig 15.

**Figure 4:**
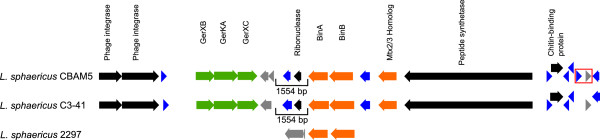
**Mosquitocidal binary toxin gene clusters of *L. sphaericus *strains CBAM5, C3-41 and 2297.** Binary toxin BinA and BinB, Mtx2/3 homolog, CDSs for a phage integrase family protein, the GerXB-KA-XC operon, a ribonuclease, a putative peptide synthase, and a chitin-binding protein, hypothetical proteins (blue arrows) and transposases (gray arrows) are indicated. A 1554 bp insertion is located between the GerXB-KA-XC operon and BinA-B coding sequences. A disrupted CDS (red box) includes a mobile element and a hypothetical protein.

The binary toxin genes *binA* (EWH32662) and *binB* (EWH32663) of *L. sphaericus* CBAM5, which are the main source of its larvicidal activity [51], were found in the contig 14 following a similar arrangement as the 35-kb duplicate cluster of *L. sphaericus* C3-41 (Figure 4). Nearby the *binA* and *binB* genes, we found a putative Mtx2/3 toxin (EWH32665), two CDSs for phage integrases in the 5’ start of the 35-kb fragment. *L. sphaericus* CBAM5 also share a germination gene cluster equivalent to the *B. anthracis* plasmid pXO1 and the BinA/BinB cluster of *L. sphaericus* C3-41, having a GerXB-KA-XC gene cluster upstream of a transposase [51,53]. Comparing the region comprised between the germination operon and the *binA-binB* genes across the sequences of *L. sphaericus* CBAM5, C3-41 and 2297, we found an equivalent homology of putative transposases with different length and disruption points. The strain CBAM5 has two mobile elements of 459 and 312 bp in length, which is similar to strains 2297 and CBAM5 showing a probable transposase pseudogene with 1,110 bp and 591 bp in length, respectively (Figure 4). As a final remark, in the 3’ end of the 35-kb fragment we found an incomplete encoding sequence for β-carotene 15,15’-monooxygenase probably disrupted by a mobile element (depicted with a red box in Figure 4). Hu et al. [51] hypothesized that the conserved 35-kb sequence, including the BinA, BinB, and the two phage integrase family proteins, are probably unique to the taxonomic *L. sphaericus* group 1 (formerly known as group IIA) being the remnant of a potential phage infection. Even though we cannot confirm the presence of additional BinA-B CDS sequences in the genome of *L. sphaericus* CBAM5, we suggest further research to confirm the participation of phage infections on the evolution of larvicidal toxins in the strain CBAM5*.*

### Surface (S) layer proteins and toxic metal resistances

*L. sphaericus* CBAM5 exhibits 21 CDSs described as surface (S) layer proteins or S-layer homologs in its genome. The fragment covered from EWH35069 to EWH35072 includes four CDSs encoding for a variable protein, a putative S-layer associated protein, a P60 invasion-associated protein and a N-acetylmuramoyl-L-alanine amidase. Probably the genes located in this fragment may participate in the larvicidal activity of the strain CBAM5, given that the same genes have been described as differentially expressed in virulent infections of *Lysteria monocytogenes*[54]. A total of 14 CDSs show three SLHs motifs near to the N terminal region, similarly to the *slpC* gene previously described in native strains [5]. In addition, we found two S-layer surface array proteins in the chromosomal scaffold and another in extracromosomal sequences.

A total of 64 CDSs corresponded to encoding sequences involved in responses against toxic metal(oid)s. Among those coding sequences, we found the following operons: *arsRBCDA, arsRBC, cadCA, mntABCD, nikABCDE, terD-terD-terD, zurR-znuBC,* and *czrA-czcD-csoR-copZA.* We could identify various genes probably involved in metal(loid) resistances spread across the genome (Additional file 4: Table S2). The *chrA* gene seems to be the only representative of the *chr* operon in the genome of *L. sphaericus* CBAM5. Previous reports have shown that microorganisms bearing *chrA* homologues display highly variable resistance levels against Cr(VI) [55]. However, two superoxide dismutase putative proteins (EWH33050, EWH30224) and several CDS ascribed as flavin reductases (EC 1.5.1.29), nitroreductases (EC 1.5.1.34) and quinone reductases (EC 1.1.5.4) could cooperate in the Cr(VI) resistance, in agreement with previous reports [55,56].

Given the heavy metal resistance of *L. sphaericus* CBAM5 in polluted environments, and supported by the identification of genes in Additional file 4: Table S2, we could expect the assistance of efflux pump systems and heavy-metal resistance proteins specific to As, Sb, Ni, Zn, Cu, Cd, Te, Cr, Mn and Co. By the evaluation of coalescent models, Villegas-Torres et al. [10] proposed that *L. sphaericus* CBAM5 may have acquired the *arsC* gene through recent events of horizontal gene transfer as a possible adaptation to polluted environments. However, we found highly similar homologues of heavy metal resistance proteins of the CBAM5 strain in microorganisms isolated from non-polluted environments (i.e. *czrA-czcD-csoR-copZA*, *cadCA,* and *arsRBC* in *L sphaericus* OT4B.31 [50]). Further analysis on plasmids, prophage content, or conjugation factors may clarify the origin of resistance (as well as larvicidal) genes. Finally, based in the KEGG analysis, some predicted proteins might participate in peripheral pathways for the degradation of geraniol, chlorocyclohexane, chlorobenzene, benzoate, bisphenol, fluorobenzoate, toluene, chloroalkane, chloroalkene, naphthalene, aminobenzoate, styrene, atrazine, limonene and pinene.

## Conclusions

*Lysinibacillus sphaericus* CBAM5 was isolated from drilling mineral base oil samples at the subsurface soil level. By comparing the chromosomal sequences between *L. sphaericus* strains CBAM5 and C3-41, we identified distinctive similarities of the DNA homology group IIA. The evidence of the binary toxins allocated in a conserved cluster delimited by mobile elements, resembles a probable phage invasion in the DNA subgroup IIA of the *Lysinibacillus sphaericus* species. Along with the biological control potential given by the Mtx, Bin and cerolysin toxins, *L. sphaericus* CBAM5 displays encoding sequences for S-layer proteins and heavy-metal efflux pumps, which may confer resistance to As, Sb, Ni, Zn, Cu, Cd, Te, Cr, Mn and Co in polluted environments.

## Competing interests

The authors declare that they have no competing interests.

## Authors’ contributions

LL performed the DNA and sequencing experiments. TDP performed antibiotics, microscopy and bioinformatics analysis. All authors drafted, read and approved the final manuscript.

## Supplementary Material

Additional file 1: Table S1. Associated record according to the MIGS recommendations. Click here for file

Additional file 2: Figure S1. Light microscopy of *Lysinibacillus sphaericus* CBAM5 growth in acetate broth. (A) Gram staining of vegetative cells after 6 hours of growth. (B) Schaeffer-Fulton staining of sporulating culture after 24 hours of growth. Click here for file

Additional file 3: Figure S2. Scanning electron micrograph of *Lysinibacillus sphaericus* CBAM5. The micrograph was obtained on a JEOL JSM-5800LV (Japan) scanning electron microscope at an operating voltage of 20 kV and 10000× magnifications. Click here for file

Additional file 4: Table S2. Genes possibly involved in metal(loid) resistances identified in the genome sequence of *Lysinibacillus sphaericus* CBAM5. Click here for file
