# Supplementary figures and images for: Genome sequence and description of the mosquitocidal and heavy metal tolerant strain Lysinibacillus sphaericus CBAM5
Source: Stand Genomic Sci. 2015 Jan 20;10:2. doi: 10.1186/1944-3277-10-2 (PMC4317669; doi:10.1186/1944-3277-10-2)

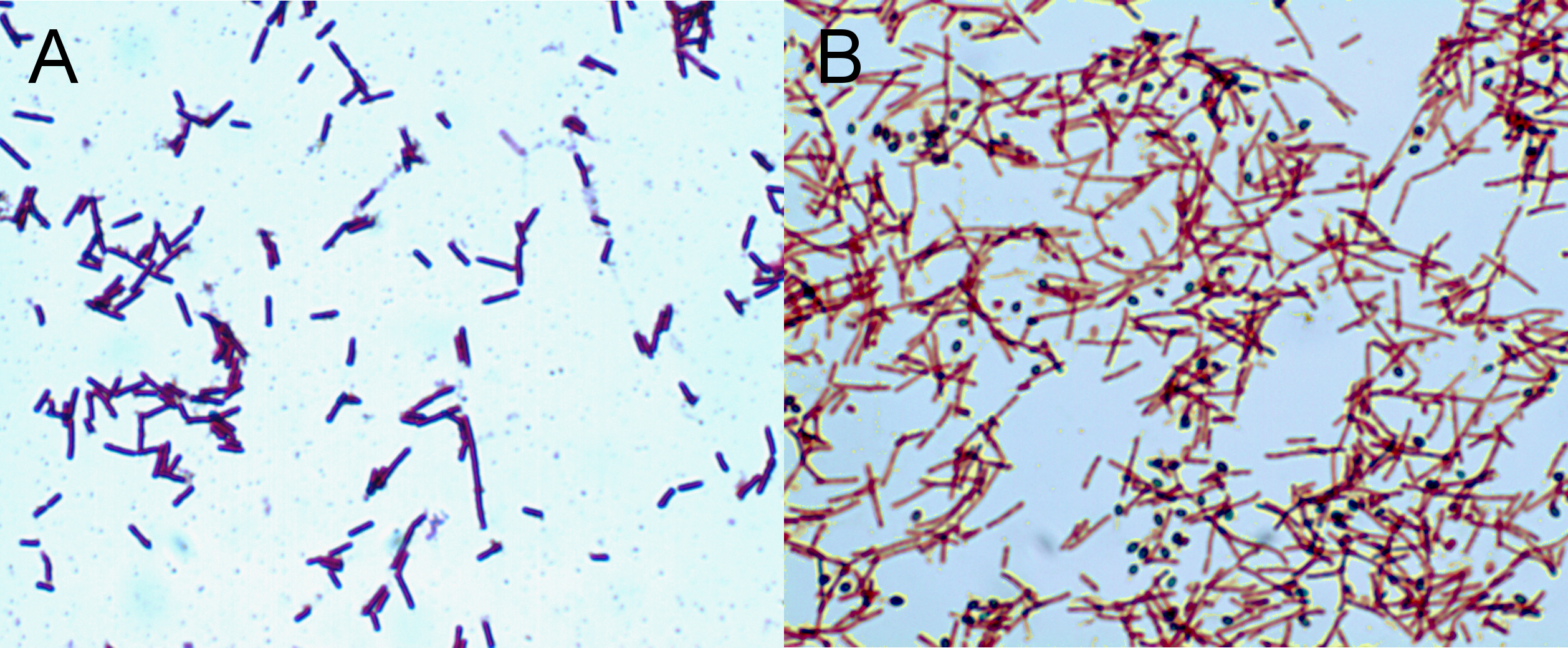

Supplement: Additional file 2: Figure S1. — Light microscopy of Lysinibacillus sphaericus CBAM5 growth in acetate broth. (A) Gram staining of vegetative cells after 6 hours of growth. (B) Schaeffer-Fulton staining of sporulating culture after 24 hours of growth. [file 1944-3277-10-2-S2.tiff]

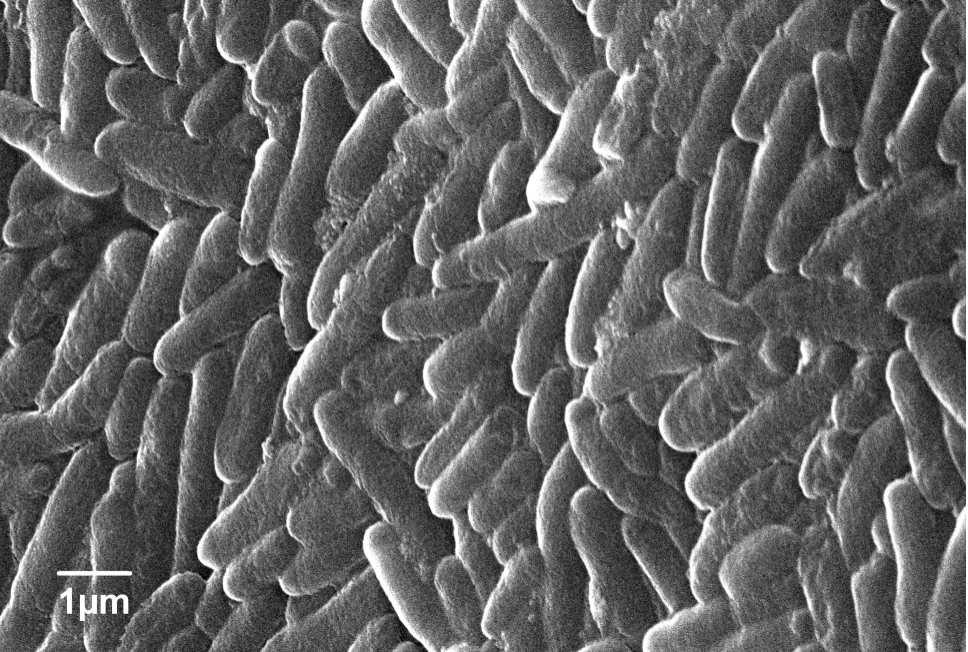

Supplement: Additional file 3: Figure S2. — Scanning electron micrograph of Lysinibacillus sphaericus CBAM5. The micrograph was obtained on a JEOL JSM-5800LV (Japan) scanning electron microscope at an operating voltage of 20 kV and 10000× magnifications. [file 1944-3277-10-2-S3.pdf]
